# Validation of B·R·A·H·M·S PCT direct, a new sensitive point-of-care testing device for rapid quantification of procalcitonin in emergency department patients: a prospective multinational trial

**DOI:** 10.1186/cc14136

**Published:** 2015-03-16

**Authors:** A Kutz, P Hausfater, M Oppert, C Alonso, C Wissmann, B Mueller, P Schuetz

**Affiliations:** 1 Kantonsspital Aarau, Switzerland; 2 Hôpital Pitié-Salpêtrière and Univ-Paris 06, Paris, France; 3 Klinikum Ernst von Bergmann, Potsdam, Germany; 4 BRAHMS GmbH, Hennigsdorf, Germany

## Introduction

Procalcitonin (PCT) is increasingly being standard in the emergency department (ED) for the diagnostic and prognostic workup of patients with suspected infections. Recently, B·R·A·H·M·S PCT direct, a new high-sensitive point-of-care test, has been developed for fast PCT measurement on capillary or venous blood samples with a measuring range of 0.1 to 10.0 μg/l.

## Methods

This is a prospective, comparative international study conducted in three European EDs. Consecutive patients with suspicion of bacterial infection were included. Duplicate determination of PCT was performed on two distinct B·R·A·H·M·S PCT direct test devices on capillary (fingertip) and venous whole blood (EDTA), and compared with the reference method (B·R·A·H·M·S PCT sensitive Kryptor or Elecsys B·R·A·H·M·S PCT, respectively). The diagnostic accuracy was evaluated by correlation and concordance analyses.

## Results

A total of 303 patients were included over a 6-month period (60.4% male, median age 65.2 years). The correlation between capillary or venous whole blood and the reference method was excellent: *r*^2 ^= 0.96 and 0.97, sensitivity 88.1% and 93.0%, specificity 96.5% and 96.8%, concordance 93% and 95% respectively at a 0.25 μg/l threshold. No significant bias was observed (-0.04 and -0.02 for capillary and venous whole blood) although there were 6.8% and 5.1% outliers, respectively. B·R·A·H·M·S PCT direct had a shorter time to result as compared with the reference method (25 vs. 147 minutes, difference 122 minutes, 95% CI = 110 to 134 minutes, *P *< 0.0001).

## Conclusion

This study found a high diagnostic accuracy and a faster time to result of the PCT direct in the ED setting. The B·R·A·H·M·S PCT direct may allow a more widespread use of PCT tests in outpatient clinics and smaller institutions.

